# Letter from O. B. Ormsby, Assistant-Surgeon, 18th Regt. Ill. Vols.

**Published:** 1863

**Authors:** O. B. Ormsby

**Affiliations:** Assistant-Surgeon, 18th Regt. Ill. Vols.; Floating Hospital, “Nashville”


FLOATING HOSPITAL, "NASHVILLE,"
*April 27th*, 1863.

DR. DAVIS,

DEAR SIR:—A long time has elapsed since my last writing; and during that time I have only had the opportunity to see the wounded of one battle,—namely, Corinth,—where I remained but seven days before I fell sick myself, and, in consequence thereof, did not write. That being now so long past, and especially as you, probably, had accounts from that field, I will not attempt to say anything about it, but give you the histories of one or two cases on the flag-boat "Nashville," where I am staying at present.

CASE I.—C. S., soldier, American, aged 25, was admitted to hospital, April 15th, as convalescent from diarrhœa: Tongue was clean; bowels regular; body emaciated; and the pulse regular, but weak. He had quinia et. ferri. citras., in small doses, simply as a desirable tonic to aid in building up his strength, and to eradicate any remains of malarious poison which might be lurking in his system. In the meantime he had permission to take exercise both on board and on shore. This course was pursued with apparent benefit until noon of the 17th. During the forenoon he had been quite active and cheerful; and when the dinner-bell rung, he remarked, that he would go and take his place at the first table. He started, and arrived at the head of the stairs, when he fell upon the floor. Word was brought me immediately, but when I reached him he was quite dead. At the autopsy, four hours after death, the rigor mortis was well-marked, the body not *extremely* emaciated, and suggillation, in dependent parts of the body, very distinct. The brain and spinal cord were exposed by the usual incisions, with no noteworthy circumstance attending, except, perhaps, that, upon division of the vertebral veins, fluid blood, to the amount of two quarts, was poured out. There was fluid contained within the membranes of the cord to the amount of, probably, f℥iv. The dura mater was, abnormally, adherent to the skull at the base of the brain, and contained about f℥ii. of effused fluid. The base of the brain was thought to be slightly softened, but, in the absence of the microscope, it could not be with absolute certainty decided. The membranes and substances of both brain and cord were considerably congested, but the nervous matter of the cord appeared to have suffered no structural lesion. The lungs were healthy. Within the pericardium was found f℥iv. or v. of serum. The muscular tissue of the heart was somewhat softened, and traces of congestion were observed. Stomach was simply congested,—liver healthy. The bowels, with the exception of the duodenum, showed evidence of nothing more than congestion of a passive character. The duodenum had, evidently, been the seat of both inflammation and ulceration; and, in several circular patches, the mucous and muscular coats were destroyed, leaving but the peritoneum. Perforation, however, had not occurred; and the ulcers seemed in process of cicatrization. The kidneys were much congested, but quite firm. This congestion appeared to have been quite recent, and to approach more nearly to an active arterial congestion than anything discovered elsewhere.

CASE II.—E. D., Frenchman, aged 25, had been admitted to hospital, April 17th, with chronic diarrhœa. He became convalescent under the use of the usual remedies; and upon the 27th was permitted to go on shore for exercise. He, however, took advantage of the opportunity to go to the sutler's store and obtain some nuts, Bologna sausages, &c., and also to strip off and bathe in the water of an adjacent bayou. During the afternoon of the 28th he was attacked suddenly with spasms, together with complete abolition of consciousness; dilated pupils; feeble and frequent pulse; and slightly stertorous breathing. Chloroform was administered with apparent benefit, and, upon the return of consciousness, also some carbonate of ammonia. He soon relapsed into another paroxysm, which was followed by a third, in which he died. Four hours after death, the body was examined. It was much emaciated,—the rigor mortis well-marked, and but little suggillation. Upon removal of the calvarium, several spots of effused lymph were discovered between the dura mater and parietal bones, together with considerable congestion of its surface. Beneath the dura mater was found two ounces of serous fluid. The brain, however, appeared to be only passively congested. The ventricles,—particularly the fourth,—were filled up with serum. The brain was unusually firm. The heart was found empty. Lungs, liver, stomach, and bowels all,—with the exception of some congestion,—healthy.

Cases of a similar character to the above have repeatedly occurred here, commencing as early as the 1st of March. As I have noticed but little intermittent here, yet I am not of the opinion that they are the result directly of miasmatic influence, though they bear, apparently, some relation to the pernicious intermittent of this latitude. The patients almost universally present marked emaciation, with great tendency to œdema and anascarca; but, aside from the debility thus indicated, very frequently do not present marked symptoms of disease. One case was of a nurse who had not been taking medicine, and had been on duty regularly. In the evening, saying, he felt unwell, he went to his bed and lay down; and, at the end of four hours, when his watch came on, he was found quite dead, and had evidently been so from one to two hours. From some cause the blood is excessively impoverished and the tissues of the body very imperfectly nourished; and, as is universally the case with debilitated subjects, congestions are very liable to occur, and to result in rapid and copious effusions.

If this is the true pathology of these cases, the important and interesting question becomes necessary: what is the cause at work which produces such a debilitated condition? It is not, certainly, in every instance disease,—in the more common acceptation of the term,—for men become so debilitated and even die off thus suddenly. Who will say, they have not "been sick, but have only run down weak?" In the solution of the question, one would, naturally, first turn to the food furnished the patients. Doing so, we find the rations ample in quantity, and of a quality sufficiently nutritious to sustain a man in vigorous health. Late physiologists, however, assert, that food, to be readily digested, should be palatable, and that the rations, *unless properly cooked*, are not.

Few of the soldiers are good cooks, and a less number still deem good cookery of sufficient importance to give it the attention which it deserves. Even the officers do not seem to understand or appreciate the fact, that food, before it is eaten, should be cooked. Cases have occurred upon this river, in which a whole regiment has been cooped up on a boat for as long a period as thirty days; and with no other chance for cooking than the boiler furnaces. Of course, the mass of the men were compelled to eat their food uncooked; and, even when the boat lies tied to the shore, still, for fear the men will get scattered, they are retained on the boat. This has been done, if not with the consent of the medical officer, at least, *not* contrary to his vigorous remonstrance; and thus the soldiers suffer a wrong, without the shadow of a chance for redress.

Probably, another great cause of the debility of these patients is, the utter listless inactivity of camp life. Troops should be compelled to take a certain amount of exercise every day; for, where this is not done, numbers will rise only upon the call to meals. Artillerists and cavalry, who have their horses to take care of, are admitted to be more healthy than infantry. Fatigue squads, who work from day to day, have only a small proportion of sick, in comparison with common soldiery.

These evils of cookery, diet, and exercise, probably, cannot be remedied in the time which our volunteer force is likely to remain in the field. Still, a proper appreciation, upon the part even of the officers, of the causes of disease, would save many a life which is now uselessly wasted. I hope and believe that the time is coming when military officers will learn that to disregard the advice of their surgeons will not promote the efficiency of their commands; and when the medical officers, feeling their responsibility for the lives and health of their men, will not hesitate to use every means in their power to promote their welfare. Yours truly, O. B. ORMSBY,

*Assistant-Surgeon*, 18*th Regt. Ill. Vols.*

